# The polymorphism rs6918289 located in the downstream region of the *TREM2* gene is associated with TNF-α levels and IMT-F

**DOI:** 10.1038/s41598-018-25553-y

**Published:** 2018-05-08

**Authors:** Vesna Gorenjak, Alex-Ander Aldasoro Arguinano, Sébastien Dadé, Maria G. Stathopoulou, Dwaine R. Vance, Christine Masson, Sophie Visvikis-Siest

**Affiliations:** 1 Université de Lorraine, Inserm, IGE-PCV, F-54000 Nancy, France; 2 Randox Laboratories Limited, Crumlin, Co. Antrim, Northern Ireland, United Kingdom; 3 Department of Internal Medicine and Geriatrics, CHU Technopôle Nancy-Brabois, Rue du Morvan, F-54511 Vandoeuvre-lès-Nancy, France

## Abstract

Triggering receptor expressed on myeloid cells 2 (TREM2) is known for its anti-inflammatory properties during the immune response, and influences negatively on TNF-α expression levels. Genetic epidemiology studies have identified polymorphisms located in the *TREM2* gene associated with neurodegenerative and chronic inflammatory diseases. TREM2 levels have been observed to affect plasma levels of TNF-α and plaque stability in symptomatic and asymptomatic patients with carotid stenosis. In this study, we investigated polymorphisms located in the *TREM2* gene region and association with TNF-α levels and the intima media thickness of the femoral artery. The discovery population from the STANISLAS Family Study comprised of 809 individuals, whereas the replication population utilized an independent cohort of French origin (*n* = 916). Our results suggest that the minor allele (T) of SNP rs6918289 is positively associated with elevated plasma levels of TNF-α in discovery and replication populations (*P* = 0.0026, *SE* = 0.04 and *P* = 0.023, *SE* = 0.09, respectively), including femoral artery thickness in the discovery cohort (*P* = 0.026, *SE* = 0.009). Results indicate that rs6918289 may be considered as a risk factor for inflammatory diseases and could be used in stratified medicine with patients diagnosed with chronic inflammatory-related conditions, such as atherosclerosis.

## Introduction

The triggering receptors expressed on myeloid cells (TREM) family molecules are members of the immunoglobulin superfamily of receptors. All five genes from the TREM family (Table 1) are situated in the 6p21.1 region of the chromosome^1^ and mediate signaling in immune cells, thus playing critical roles in inflammatory responses^2^. The region 6p21.1 is in proximity to the MHC/HLA region of the genome, which is implicated in the immune response, autoimmunity and risk of autoimmune diseases^3,4^. Specifically, the TREM2 molecule is primarily expressed on the cell surface of macrophages and dendritic cells derived from monocytes, as well as in microglia and osteoclasts. TREM2 binds to the DAP12 trans-membrane molecule and is responsible for a series of tyrosine phosphorylation reactions that regulate various inflammatory responses^2,5^. TREM2 portrays anti-inflammatory properties during the immune response^6,7^, including: stimulation of phagocytosis and suppression of cytokine production, *e.g*. TNF-α^8,9^, one of the most important molecules for the regulation of inflammation, and reflects the degree of inflammatory response.

**Table 1:** TREM family of genes.

Gene	Gene name	Chromosome
*TREML1*	triggering receptor expressed on myeloid cells like 1	6p21.1
*TREML2*	triggering receptor expressed on myeloid cells like 2	6p21.1
*TREML4*	triggering receptor expressed on myeloid cells like 4	6p21.1
*TREM1*	triggering receptor expressed on myeloid cells 1	6p21.1
*TREM2*	triggering receptor expressed on myeloid cells 2	6p21.1

It is evident that TREM2 acts as a protective molecule in chronic inflammatory diseases. For example, studies in transgenic mice have demonstrated that deficiency of TREM2 protein may accelerate the aging process, reduce microglial activity and result in neuroinflammation, which plays a major role in all neurodegenerative diseases^10^. Furthermore, atherosclerosis is a chronic cardiovascular inflammatory disease, caused by activation of the immune system mediating the chronic inflammatory process of the arterial wall. Interestingly, TREM2 has also been shown to play an important role in the stability of atherosclerotic plaques^11^.

Polymorphisms located in the *TREM2* gene have been correlated with neurodegenerative and chronic inflammatory diseases, such as Alzheimer’s disease^12,13^, frontotemporal dementia^14,15^, Parkinson’s disease^16,17^, inflammatory bowel disease^18^ and stroke^19^. However, no genetic determinants have been identified in the *TREM2* locus affecting plasma levels of TNF-α and/or intima media thickness.

Due to the role of TREM2 in the inflammatory response and stability of atherosclerotic plaques, we hypothesize that polymorphisms in the *TREM2* gene region may influence the inflammatory process and subsequent atherosclerotic plaque formation. In this investigation, we have studied the association of variants located in the *TREM2* gene regionwith plasma levels of TNF-α and intima media thickness of the femoral artery (IMT-F).

## Results

Information on the genotyped polymorphisms as well as the demographic and clinical characteristics of the studied populations is shown in Tables 2 and 3, respectively. All SNPs analyzed within this investigation were in agreement with Hardy-Weinberg equilibrium (*P* > 0.001).

**Table 2:** Characteristics of the genotyped polymorphisms in *TREM 2* region.

Children STANISLAS Family Study (SFS)					
SNP	Gene	Minor allele	MAF	Chromosome	HWE *P*
rs7748777	*LOC105375056*	A	0.44	6p21	1
rs6918289	*ADCY10P1*	T	0.13	6p21	0.46
rs7759295	*LOC105375056*	T	0.11	6p21	1
rs9357347	*LOC107986595*	C	0.29	6p21	1
rs6915083	*TREML2*	G	0.38	6p21	0.28
**Total SFS**					
rs6918289	*ADCY10P1*	T	0.13	6p21	0.73
**Replication population (Adults)**					
rs6918289	*ADCY10P1*	T	0.12	6p21	0.89

With the exception of the SNP rs6918289, the rest were only genotyped in children.

**Table 3:** Demographic and clinical characteristics of studied populations.

	SFS Children	SFS Total	SFS with IMT-F	Replication Population
Sample size [% female]	139 [52.5%]	809 [48.7%]	350 [53.1%]	916 [50.8%]
Age (years) [S.D]	15.4 [2.19]	30.3 [14.10]	31.9 [14.86]	55.5 [11.15]
BMI (kg/m²) [S.D]	19.9 [2.22]	22.9 [3.99]	23.2 [4.19]	26.6 [3.47]
TNFa	3.57 [2.18]	3.26 [2.13]	—	2.68 [0.94]
IMT-F	—	—	0.50 [0.06]	—

### Genetic association of SNPs in *TREM2* gene region with TNF-α concentration

Firstly, association analysis was performed in 139 children from the STANISLAS Family Study (SFS). SNPs that were located and previously genotyped in the *TREM2* gene region were tested for association with TNF-α concentration. Among the five SNPs studied, the minor allele (T) of rs6918289 was significantly associated with increased TNF-α concentrations (*P* = 0.0015, Table 4).

**Table 4:** Genetic association of the SNPs in *TREM2* region with TNF-α levels in children of the SFS (*n* = 139).

Polymorphism	Model	P-value	Beta	S.E
rs7748777	Additive	0.04014	0.14	0.07
rs6918289	Additive	**0.00147**	0.33	0.10
rs7759295	Additive	0.9966	−0.0005	0.11
rs9357347	Additive	0.02127	0.17	0.075
rs6915083	Additive	0.1634	0.1064	0.076

P-value threshold is ***P* < 0.01**. Significant p-values are highlighted in bold.

**Figure 1:**
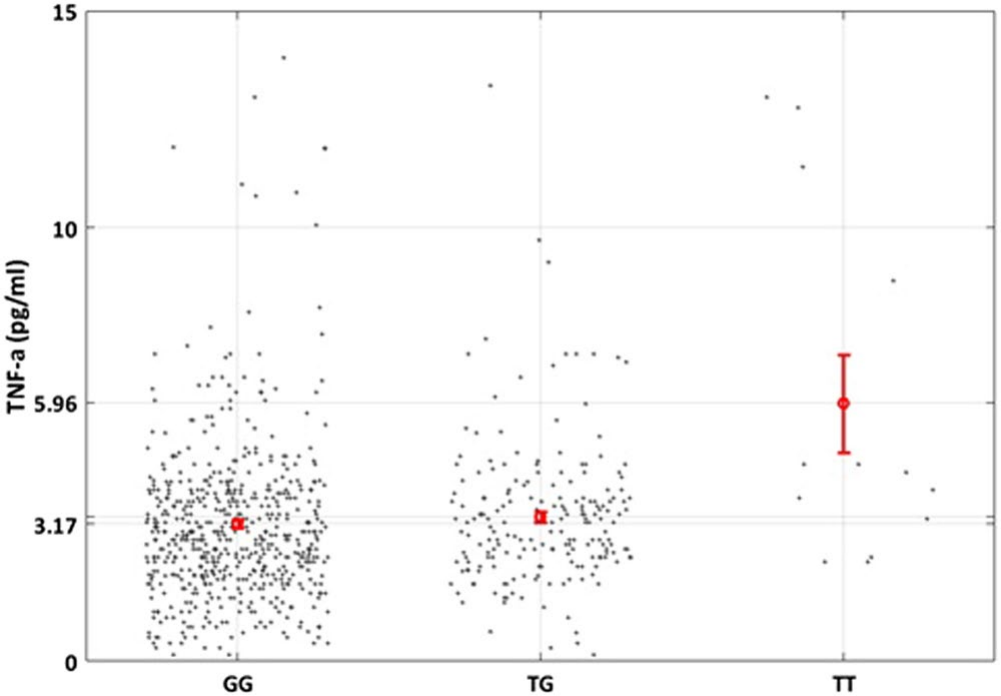
Mean values of TNF-α levels according to the different genotypes of rs6918289 (GG vs TG vs TT) in the SFS population. Thin bars show standard errors.

Secondly, rs6918289 was genotyped in 393 adult and additional 277 children relatives from the SFS. Analysis was performed in the combined population of SFS (*n*_*total*_ = 809) using three genetic models. The additive genetic model showed a positive association between polymorphism rs6918289, located in the *TREM2* gene downstream region and TNF-α plasma levels (*P = *0.0026, β = 0.13, Table 5) (Fig. 1). The recessive model showed stronger genetic association with TNF-α plasma levels (*P* = 0.0017, β = 0.498, Table 5).

**Table 5:** Association analysis of the polymorphism rs6918289 with TNF-α levels in the discovery and replication populations.

Population	Genetic model	N	Beta	SE	P-value
SFS	**Additive**	809	0.13	0.04	**0.0026**
	**Dominant**	809	0.11	0.05	**0.022**
	**Recessive**	809	0.49	0.15	**0.0017**
Replication population	**Additive**	916	0.042	0.02	0.073
	**Dominant**	916	0.03	0.03	0.189
	**Recessive**	916	0.20	0.09	**0.023**

P-value threshold is ***P* < 0.05**. Significant p-values are highlighted in bold.

Further association analysis was performed in an independent French population of European ancestry, consisting of 916 individuals. However, only a marginal association for the additive model was observed (*P* = 0.073, β = 0.042, Table 5). A significant association was observed for the recessive model (*P* = 0.023, β = 0.202, Table 5). Interestingly, the minor allele (T) of SNP rs6918289 was associated with elevated levels of TNF−α in the discovery and replication populations.

Meta-analysis of the SFS and the replication populations revealed similar results: A marginal association with TNF-α for the additive model results (*P* = 0.072) that becomes significant in the recessive model (*P* = 0.0003).

### Genetic association of rs6918289 with IMT-F

**Figure 2:**
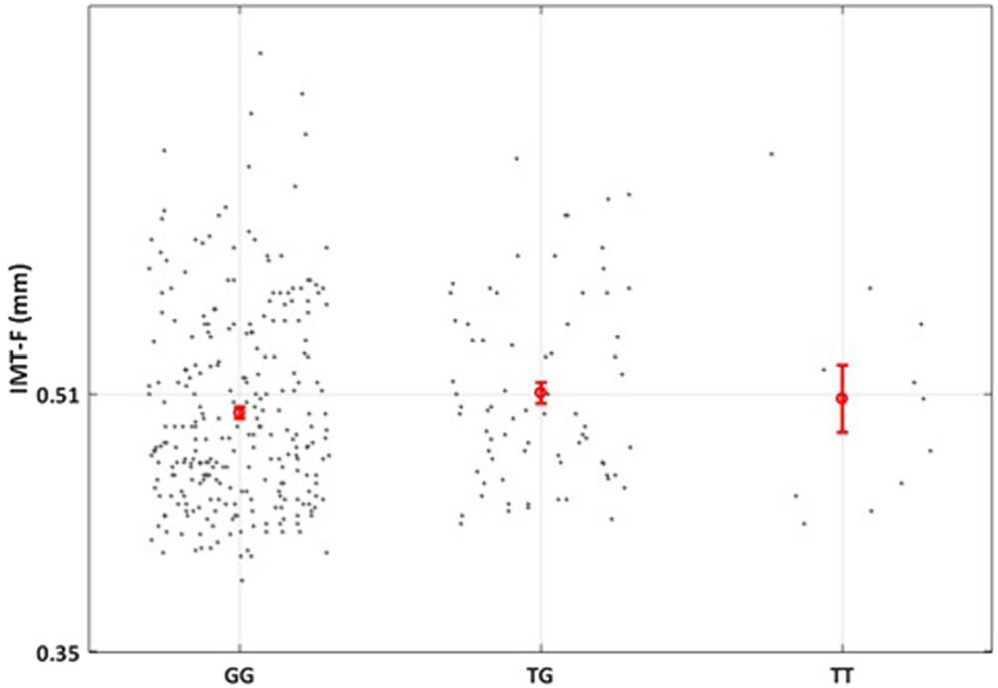
Mean values of intima media thickness of IMT-F according to the different genotypes of rs6918289 (GG vs TG vs TT) in the SFS population. Thin bars show standard errors.

A sub-group of 350 individuals, consisting of adults and children from the SFS population, where IMT-F measurements were available, was used to identify association with SNP rs6918289. The additive genetic model showed significant association (*P* = 0.026, β = 0.02, Table 6). The association was also significant for the dominant model (*P* = 0.026, β = 0.024, Table 6). Thus, the minor allele (T), of SNP rs6918289 was associated with increased thickness of the femoral artery (Fig. 2).

**Table 6:** Association analysis of the polymorphism rs6918289 with IMT-F in the discovery population (not available in the replication population).

Population	Genetic model	N	Beta	SE	P-value
SFS	**Additive**	350	0.02	0.009	**0.026**
	**Dominant**	350	0.024	0.01	**0.026**
	**Recessive**	350	0.024	0.025	0.34

P-value threshold is ***P* < 0.05**. Significant p-values are highlighted in bold.

### Bioinformatics analyses

#### Polymorphism location

SNP rs6918289 is located on chromosome 6 in p21.1 region. Located at 41 134 089 bp, it is an intron variant of adenylate cyclase 10 pseudogene 1 (ADC10P1) (Sup. Figure 1). The polymorphism on the forward strand is G > T with a minor allele frequency of 0.07 for thymine in 1000 Genome project^20,21^.

#### Phylogenetic context

The guanine polymorphism of rs6918289 G > T is evolutionary and well-conserved in primates and mammals in general (Sup. Table 1). Further phylogenetic studies performed in 33 mammals and 46 vertebrates (Sup. Figure 2) showed that this polymorphism has a slower evolution rate than expected, having a PhyloP score of 0.061 and 0.056 respectively. This strengthens the idea that this variant is involved in important molecular mechanisms and that its preservation has been sustained throughout the natural selection processes.

#### Genomic context

SNP rs6918289 variant overlaps 2 transcripts and is located between two terminal exons of *ADCY10P1*: *ADCY10P1-202* (3785 bp - between exon 17 and 18) and *ADCY10P1-203* (4569 bp - between exon 18 and 19), both leading to transcripts that are not translated into proteins (Sup. Figure 2). Furthermore, according to the expressed sequence tags database (dbEST, https://www.ncbi.nlm.nih.gov/dbEST) available in Ensembl, the polymorphism rs6918289 is located within an intense transcriptionally active locus (Sup. Figure 3)^20,21^.

## Discussion

The anti-inflammatory effects of TREM2 have been described in several studies. Indeed, knockdown or silencing of *TREM2* gene results in increased levels of different pro-inflammatory molecules, among them TNF-α^6,7,22^. Despite the fact that several polymorphisms within the *TREM2* gene have been related with numerous neurodegenerative and inflammatory diseases^12,16,18,19^, to date, no genetic determinants have been identified in the *TREM2* locus (6p21.1) affecting TNF-α concentrations.

In this study, we evaluated the effects of SNPs located in the *TREM2* gene region on TNF-α concentrations and IMT-F measurements. For 139 children from the SFS population, genotypes for five SNPs (rs7748777, rs6918289, rs7759295, rs9357347 and rs6915083) located in the TREM2 gene region were readily available. After conducting association analysis for the five aforementioned SNPs with TNF-α concentrations, we observed the minor allele (T) of SNP rs6918289, located in the downstream region of the *TREM2* gene, to be associated with elevated levels of TNF-α (*P* = 0.0003). Importantly, further analysis in a larger sample of SFS population, as well as replication in an independent population and a meta-analysis of discovery and replication populations confirmed this novel association. Subsequently, we also performed association analysis of rs6918289 with intima-media thickness of the femoral artery in 350 individuals of the discovery population. Results suggest that the minor allele (T) of SNP rs6918289 is associated with increasing intima-media thickness of the femoral artery. Therefore, this is evidence that the minor T allele may be considered as a risk allele for inflammatory diseases and atherosclerosis.

The role of both studied phenotypes in the development of atherosclerosis and the prognosis of atherosclerotic patients is well documented. TNF-α is a key regulator of immune response and alterations of its levels lead to elevated inflammation and a subsequent deterioration of the outcome of patients with cardiovascular diseases^23,24^. Also, one of the early processes that lead to atherosclerosis is the arterial remodeling and one effective way that provides information about this process is measuring the intima-media thickness. Indeed, IMT is predictive of atherosclerosis in asymptomatic individuals^25,26^, and also provides information about the degree of atherosclerosis^27,28^ as well as predicting the future risk of suffering a myocardial infarction event^29^.

Chromosomal 6p21.1 genetic region, which is in proximity to the MHC and HLA regions, has been highly studied, and associated with autoimmune diseases^3,4^. Interestingly, previous GWAS studies have associated polymorphisms located within this genetic region with atherosclerotic stroke^30^. Although SNP rs6918289 is not in linkage disequilibrium with the variants associated with atherosclerotic stroke (rs556621 and rs556512), our results strengthen the idea that chromosomal 6p21.1 region could, indeed, be correlated with atherosclerosis risk. Our results are providing new insights on possible genetic regulations of pathological pathways that could lead to increased risk of atherosclerosis.

Despite the fact that this polymorphism contributes novel insights about its potential role in the development of cardiovascular diseases, the exact mechanisms by which this process is orchestrated is so far unknown and further studies are warranted. Indeed, there are no previous studies that associate this SNP or SNPs in linkage disequilibrium with TNF-α levels or IMT-F. In support of our findings, previous investigations suggest that TREM2 affects plasma TNF-α concentrations^7–9,22^. Also, our bioinformatic analyses indicate that rs6918289 is located in a transcriptional region of *ADC10P1* gene that could impact TREM2 transcription levels^31^. Indeed, rs6918289 is referred by rSNPBASE (http://rsnp.psych.ac.cn/) as a post-transcriptional regulatory element and, in lymphocyte B cells, rs6918289 is associated with the PABPC1 protein. The PABPC1 protein binds to the 3′ poly(A) region of the mRNAs. Although the binding of this protein is necessary for the translation initiation, it is also required for poly(A) shortening, which is the first step in mRNA decay^32^. Thus, the minor allele (T) of SNP rs6918289 could affect the PABPC1 protein, which would at the same time affect the stability of the TREM2 mRNA, consequently contributing to elevated plasma TNF-α concentrations.

**Figure 3:**
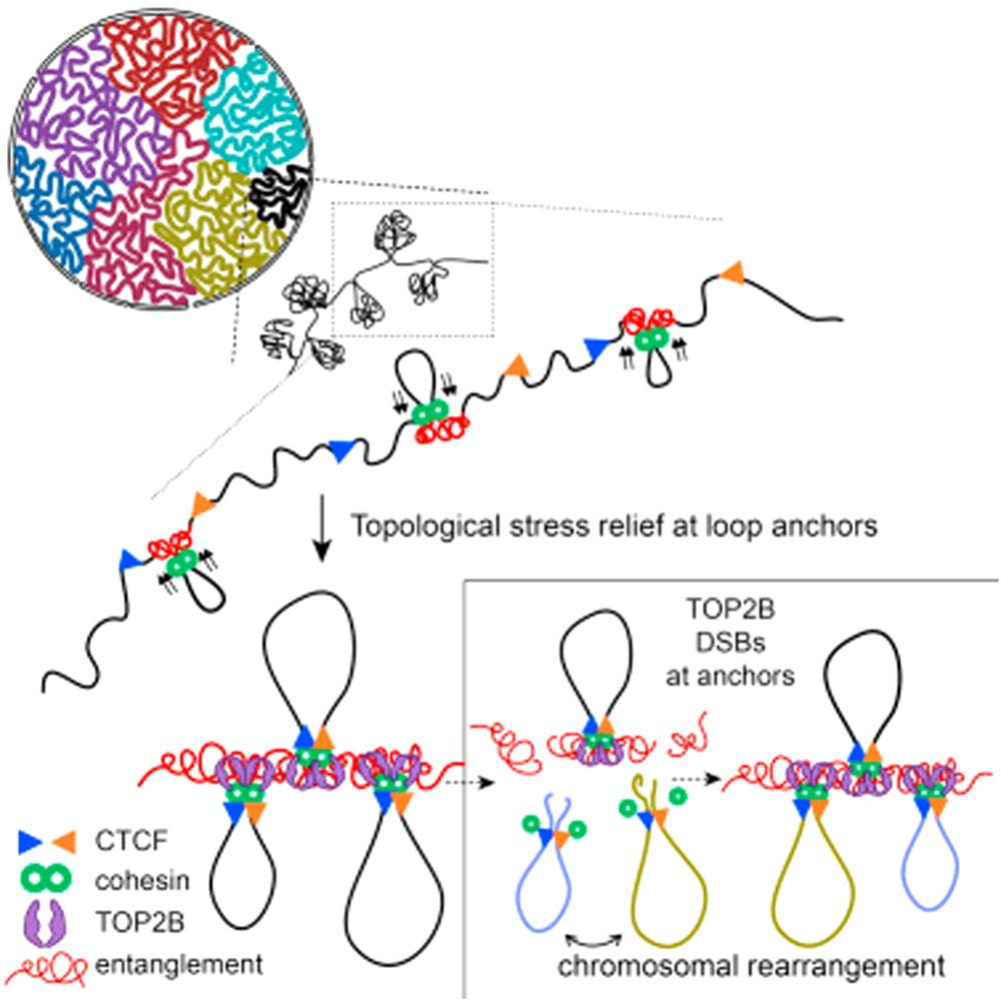
Organization of DNA forming topologically associating domain (TAD) within two CTCF sites in DNA sequence. (Retrieved from http://www.cell.com/cell/fulltext/S0092-8674(17)30718-3).

An alternative or synergistic mechanism is also conceivable. As shown by several studies, many RNAs bind to transcriptional repressor CTCF (11-zinc finger protein) to modulate its regulatory functions^33^. SNP rs6918289 is located between two CTCF genomic sequences that are “together” (Sup. Figures 2 and 3), which means they are forming a chromatin loop leading to a topologically associating domain (TAD) (Fig. 3).

TAD is a large, megabase-sized local chromatin interaction domain that forms a self-interacting genomic region^34^. The structural and organizational changes of such region affect gene expression and other cellular functions, such as re-organization of local interactions between enhancers and promoters. Moreover, DNA sequences within a TAD physically interact with each other more frequently than with sequences outside the TAD^35^. Thus, we hypothesize that CTCF, in association with the non-coding RNAs of *ADCY10P1*, could affect TREM2 expression levels. The minor allele (T) of SNP rs6918289 could promote the binding of CTCF and consequently trigger an insulation mechanism for *TREM2* gene. These hypotheses should be tested in future works in order to conclude about the involved mechanisms.

Finally, the rs6918289 can be found in proximity to the *TREML1* gene. *In silico* analysis showed significant correlation of variant with seven SNPs related to TREML1 gene (Sup. Table 2). Therefore, *TREML1* could also be a possible mediator of the effect on plasma TNF-α concentration. *TREML1* gene (Triggering Receptor Expressed On Myeloid Cells Like 1) encodes a protein, involved in platelet aggregation, inflammation, and cellular activation. Together with TREM 2 they are involved in common pathways in the setup of Alzheimer’s disease^36^.

Concerning the IMT-F results, we think that the effect of SNP rs6918289 could be indirect. Indeed, the remodeling of the artery is highly dependent on the inflammatory state, thus, increased TNF-α levels produced by the minor allele (T) of SNP rs6918289 could be responsible for the increased thickness of the femoral artery. Further studies will be necessary in order to clarify if this polymorphism or polymorphisms in linkage disequilibrium are capable of modulating the TREM2 protein levels and to discover which are the mechanisms behind this observation. However, important applications could be identified as these findings may be used for personalized treatments in patients with chronic inflammatory diseases^37^.

Limitation of our study is the unexplained mechanism of the rs6918289 effect on TNFα levels. Nevertheless, few pertinent hypotheses were proposed and future advances in genomic research with advanced bioinformatics tools will enable more precise explications of genetic interactions. Finally, genetic ancestry was not tested for populations used in this study; however strict inclusion criteria for origin were set aiming to collect homogeneous population, which are adequate for genetic association studies.

In summary, our study indicates variant rs6918289 located in the downstream region of the TREM2 gene as a candidate risk factor for inflammatory diseases because of its tight association with plasma TNF-α concentration. These findings support results of previous studies linking variants within chromosomal 6p21.1 loci to atherosclerosis and thus raising the awareness to consider genomic region 6p21.1 as candidate susceptibility loci for atherosclerosis.

## Methods

### Ethics statement

The samples are part of a human sample storage platform: BRC IGE-PCV number BB-0033–00051 located in Nancy, East France. All participants provided written informed consent. All populations involved in this study were recruited in accordance with the latest version of the Declaration of Helsinki for Ethical Principles for Medical Research Involving Human Subjects. All the protocols were approved by the local ethics committees for the protection of subjects for biomedical research: the Comité Consultatif de Protection des Persones dans la Recherche Biomédicale (CCPPRB).

### Study participants

The discovery and replication populations are part of the Biological Resources Center ‘Interactions Gène-Environnement en Physiopathologie CardioVasculaire’ (BRC-IGE-PCV, number BB-0033–00051) in Nancy, France.

Individuals, comprising children and adults from the SFS^38^ were used as the discovery population. The SFS include more than 1,000 nuclear families, each composed of at least four individuals (two parents and two children). All families are of French origin (parents and grandparents of French origin and residence in the Lorraine region) and were recruited at the Centre for Preventive Medicine of Vandoeuvre-lés-Nancy (East France). Even though strict criteria of French (European) origin were set for the inclusion of individuals in both populations, genetic ancestry was not tested. All individuals were free of chronic diseases. Firstly, we used samples from 139 children of the SFS, where genotype information was readily available for the *TREM2* gene region, to conduct association studies with plasma levels of TNF-α. Subsequently, 393 adult samples and 277 additional children samples from the corresponding families were included, reaching a total of 809 participants.

The replication cohort included 916 unrelated adults of French origin. Their inclusion criteria were the same as in the discovery cohort.

### Blood samples and biological measurements

Blood samples were taken from individuals after an overnight fast (>8 hours). The plasma concentrations of TNF-α were measured by a commercially available enzyme-linked immunosorbent assay (ELISA) (R&D Systems, UK) according to manufacturer instructions. Body mass index (BMI) was calculated as weight divided by height squared (Kg/m²). IMT-F was measured in 350 SFS individuals (including children and adults) using B-mode ultrasound methods^39^. The right and left femoral arteries were examined with a 7.5 MHz probe, according to a protocol already described^40,41^. For each individual, two IMT-F measurements were obtained and right and left measurements were used to calculate the mean IMT-F (in mm). IMT-F data were not available in the replication population.

### Genotyping

Genome-wide genotypes were readily available for all children samples from the SFS (*n* = 139). Genotyping was performed using Illumina® human CNV370-Duo array^42^. The Illumina® protocol for the BeadStation genotyping platform was used, followed by GenCall® software analysis to automatically collect, call genotypes, and designate confidence scores using the GenTrain clustering algorithm. The selection of the SNPs was done by first extracting from the genome-wide assay all the SNPs available in the region around *TREM2* gene locus (50 kb upstream and downstream of the *TREM2* gene). We employed PLINK software^43^ to conduct this analysis that resulted in the extraction and selection of five SNPs.

After performing initial association analysis in the child cohort of the SFS, polymorphism rs6918289 was *de novo* genotyped in the adult population (*n* = 393) and children population (*n* = 277) of SFS and the replication population (*n* = 916). Genotyping of rs6918289 in the replication population was conducted by Laboratory of the Government Chemist (LGC), using a PCR-based KASP assay^44^.

### Statistical analysis

Normal distribution was tested by Kolmogorov-Smirnov test. If phenotypes did not conform to normal distribution, data were log transformed in order to reach normality. The Hardy-Weinberg Equilibrium (HWE) was tested using the chi-square test (*P* > 0.001). The SNP effects on the studied phenotypes were tested through linear regression adjusted for age, gender and BMI under three inheritance models (additive, dominant and recessive) and using the minor allele as the reference allele. Analyses were performed using the R package GWAF (Genome-Wide Association/Interaction Analysis and Rare Variant analysis with Family Data)^45^ taking into account familial resemblance. Alternatively, population characteristics were determined using SPSS statistical software version 20.0 (SPSS, Inc., Chicago, Illinois). Firstly, we tested the association of five SNPs located in the *TREM2* gene region that were extracted from the GWAS available in children of the SFS (*n* = 139) with plasma TNF-α concentrations. Secondly, we tested the significance of SNP rs6918289 with TNF-α plasma levels in all available individuals of SFS (children and adults combined; *n* = 809) and in the replication population (*n* = 916). Meta-analysis of the discovery and replication cohorts was performed using the GWAMA software and a random-effect method. Thirdly, in 350 individuals of the SFS, rs6918289 was tested for association with IMT-F. Bonferroni correction was applied in order to adjust for multiple testing. The P-value threshold was set at *P* < 0.05/5 = 0.01 for the first analysis made in children, *P* < 0.05/1 = 0.05 for the analysis made in all individuals of the discovery population (children and adults) and *P* < 0.05/1 = 0.05 in the replication population and the meta-analysis.

### Bioinformatics analysis

Location, genomic and phylogenetic context of rs6918289 were determined on the Human genome (GRCh38.p10) using Ensembl browser^20^. The putative regulatory role of rs6918289 was established using rSNPBASE^21^, and the PhyloP score was obtained using PhyloP software^46^.

### Data availability

Extensive data is provided with this article and further information is available from the authors on request.

## Electronic supplementary material

Supplementary data
