# Supplementary material for: The polymorphism rs6918289 located in the downstream region of the TREM2 gene is associated with TNF-α levels and IMT-F
Source: Sci Rep. 2018 May 8;8:7160. doi: 10.1038/s41598-018-25553-y (PMC5940861; doi:10.1038/s41598-018-25553-y)
Supplement: Supplementary file 1 — Supplementary data [file 41598_2018_25553_MOESM1_ESM.doc]

SUPPLEMENTARY INFORMATION

The polymorphism rs6918289 located in the downstream region of the *TREM2* gene is associated with TNF-α levels and IMT-F

Vesna Gorenjak^1^*, Alex-Ander Aldasoro Arguinano^1^*, Sébastien Dadé^1^, Maria Stathopoulou^1^, Dwaine R.Vance^2^, Christine Masson^1^, Sophie Visvikis-Siest^¥, 1, 3^

^1^ Université de Lorraine, Inserm, IGE-PCV, F-54000 Nancy, France

^2^ Randox Laboratories Limited, Crumlin, Co. Antrim, Northern Ireland, United Kingdom

^3^ Department of Internal Medicine and Geriatrics, CHU Technopôle Nancy-Brabois, Rue du Morvan, F-54511, Vandoeuvre-lès-Nancy, France

***Equal first authors**

**^¥^Corresponding author:**

Dr. Sophie VISVIKIS-SIEST

INSERM UMR U1122; IGE-PCV, Faculté de Pharmacie – Université de Lorraine

30 rue Lionnois

54000 Nancy, France

Tel: +33.6.07.60.25.69

E-mail address: [sophie.visvikis-siest@inserm.fr](mailto:sophie.visvikis-siest@inserm.fr)

**Sup. Figure 1**: Location and genomic context of rs6918289 on chromosome 6 in p21.1 region (41 134 089 bp).


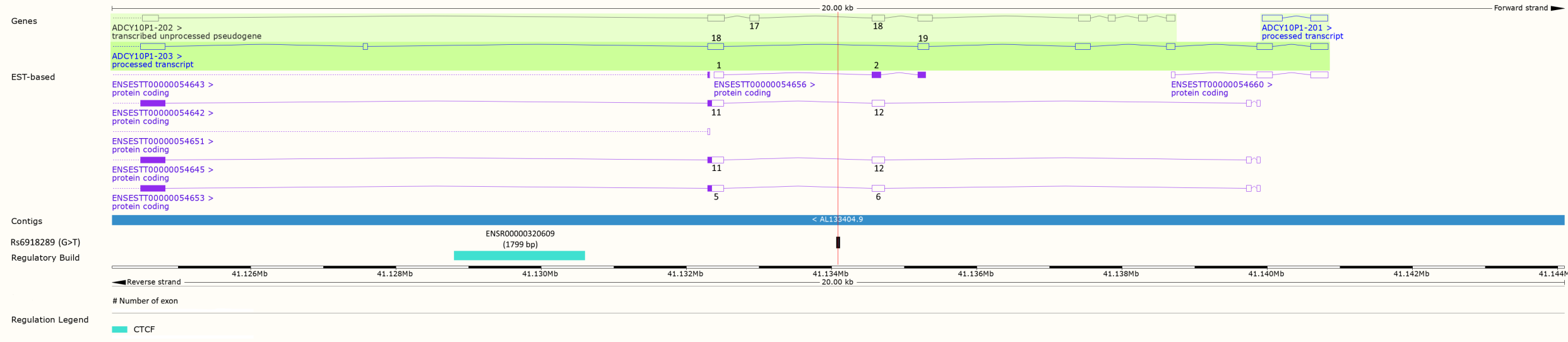


According to the expressed sequence tags database (dbEST) available in Ensembl, the polymorphism rs6918289 is located within an **intense transcriptionally active locus**. Apart of the gene ADY10P1, it also belongs to *ENSESTG00000024983* gene. This gene has 7 transcripts and among them *ENSESTT00000054656,* which is translated into a 77 amino acids protein and contains rs6918289 between exon 1 and 2; the *ENSESTT00000054642*, which is translated into a 262 amino acids protein and contains rs6918289 between exon 11 and 12; the *ENSESTT00000054645,* which is translated into a 262 amino acids protein and contains rs6918289 between exon 11 and 12; and the *ENSESTT00000054653,* which is translated into a 262 amino acids protein and contains rs6918289 between exon 5 and 6. A CTCF binding site, the *ENSR00000320609*, is located at 3489 bp in the 5’ direction from rs6918289. Another CTCF binding site, the *ENSR00000320611,* is also present, located at 26015 bp from *TREM2* gene, in the 5’ direction (Sup. Figure 3).

**Sup. Table 1: Guanine conservation of rs6918289 through primates and one eutherian mammal.**

| **Specie** | **Sequence** | **Location** |
| --- | --- | --- |
| **Human** | TCACTTTCTTGAGGGATTAG | CRCh38:6:41134079:41134099:1 |
| **Chimpanzee** | TCACTTTCTTGAGGGATTAG | CHIMP2.1.4:6:41696034:4169654:1 |
| **Gorilla** | TCACTTTCTTGAGGGATTAG | gorGor3.1:6:42328798:42328818:1 |
| **Orangutan** | TCACTTTCTTGAGGGATTAG | PPYG2:6:41219530:41219550:1 |
| **Vervet-AGM** | TCACTTTCTTGAGGGATTAG | ChlSab1.1:17:31023954:31023974:-1 |
| **Macaque** | TCACTTTCTTGAGGGATTAG | Mmul_8.0.1:4:42085379:42085399:1 |
| **Olive baboon** | TCACTTTCTTGAGGGATTAG | PapAnu2.0:4:40443409:40443429:1 |
| **Marmoset** | TTACTTTCTTGAGGGATTAG | C_jacchus3.2.1:42316089:42316109:1 |
| **Rabbit** | TCACTTTCTTGGTGGGTCTGT | OryCun2.0:12:30888427:30888447:1 |

**Legend:** Focus variant; Differs from primary species

**Sup. Figure 2**: PhyloP scores of 46 vertebrates and 33 mammals for the guanine of rs6918289.


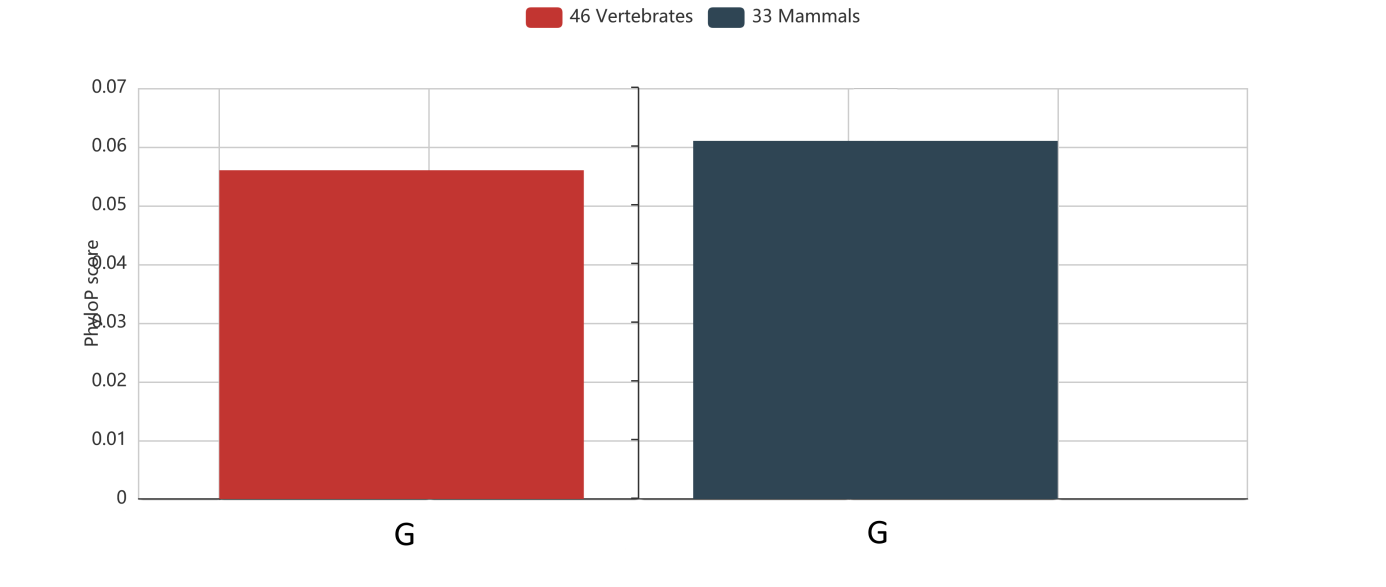


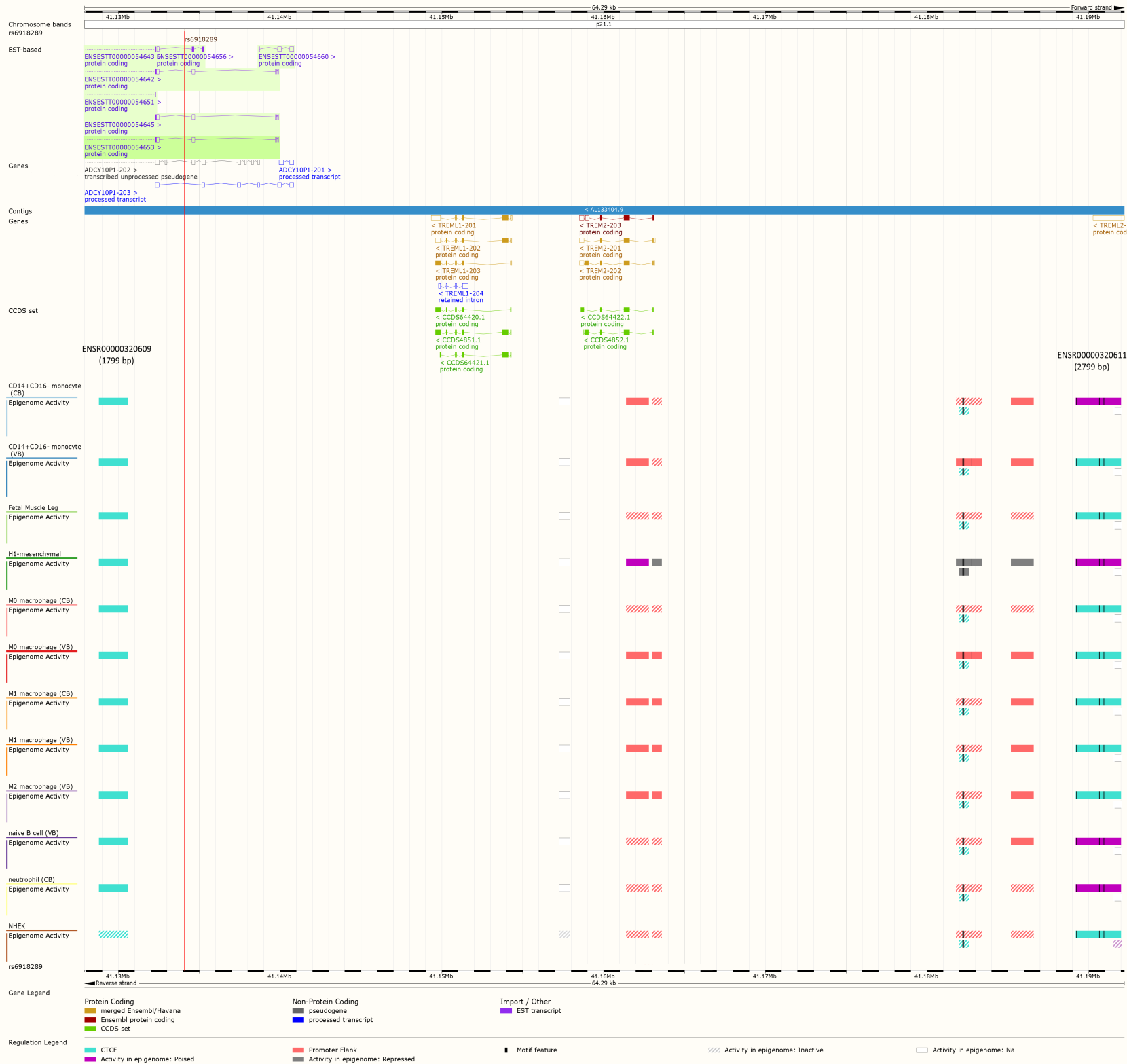
**Sup. Figure 3:** Regulatory environment of rs6918289 and *TREM2* gene.

These two CTCF binding sites are together active (In turquoise) or poised (in purple) in only 6 cell types over the 23 studied: monocyte (CB and VB), neutrophil (CB), fetal muscle leg, H1-mesenchymal, macrophage M0, M1 (CB and VB) and M2 (VB), and naïve B cell (VB).

**Sup. Table 2:** Variants related to rs6918289 in TREM region.

| **CHR** | **Pos (hg38)** | **LD (r^2^)** | **Variant** | **Ref** | **Alt** | **Motifs changed** | **GENCODE genes** | **dbSNP func annot** |
| --- | --- | --- | --- | --- | --- | --- | --- | --- |
| **6** | 41145639 | 1 | [rs62396347](http://archive.broadinstitute.org/mammals/haploreg/detail_v4.1.php?query=&id=rs62396347) | G | A | Mef2 | 3.6kb 3' of TREML1 | - |
| **6** | 41145790 | 1 | [rs62396348](http://archive.broadinstitute.org/mammals/haploreg/detail_v4.1.php?query=&id=rs62396348) | T | G | 4 altered motifs | 3.5kb 3' of TREML1 | - |
| **6** | 41146182 | 1 | [rs72856298](http://archive.broadinstitute.org/mammals/haploreg/detail_v4.1.php?query=&id=rs72856298) | T | C | SIX5,TBX5 | 3.1kb 3' of TREML1 | - |
| **6** | 41150086 | 0.95 | [rs45537633](http://archive.broadinstitute.org/mammals/haploreg/detail_v4.1.php?query=&id=rs45537633) | G | A | CAC-binding-protein,Ik-1 | TREML1 | intronic |
| **6** | 41151782 | 0.97 | [rs62396351](http://archive.broadinstitute.org/mammals/haploreg/detail_v4.1.php?query=&id=rs62396351) | G | C | GR | TREML1 | intronic |
| **6** | 41153296 | 0.97 | [rs62396352](http://archive.broadinstitute.org/mammals/haploreg/detail_v4.1.php?query=&id=rs62396352) | G | C | CTCF,Rad21 | TREML1 | intronic |
| **6** | 41153359 | 0.97 | [rs62396353](http://archive.broadinstitute.org/mammals/haploreg/detail_v4.1.php?query=&id=rs62396353) | G | A |  | TREML1 | intronic |

The analysis of functional inference with HaploReg (<http://archive.broadinstitute.org/mammals/haploreg/haploreg.php>) indicated one hundred significant variants (r2 >= 0.8 with rs6918289). Seven detected variants were related to TREML1 gene.

**Sup. Figure 4:** UCSC Genome Browser analysis of rs6918289 region.


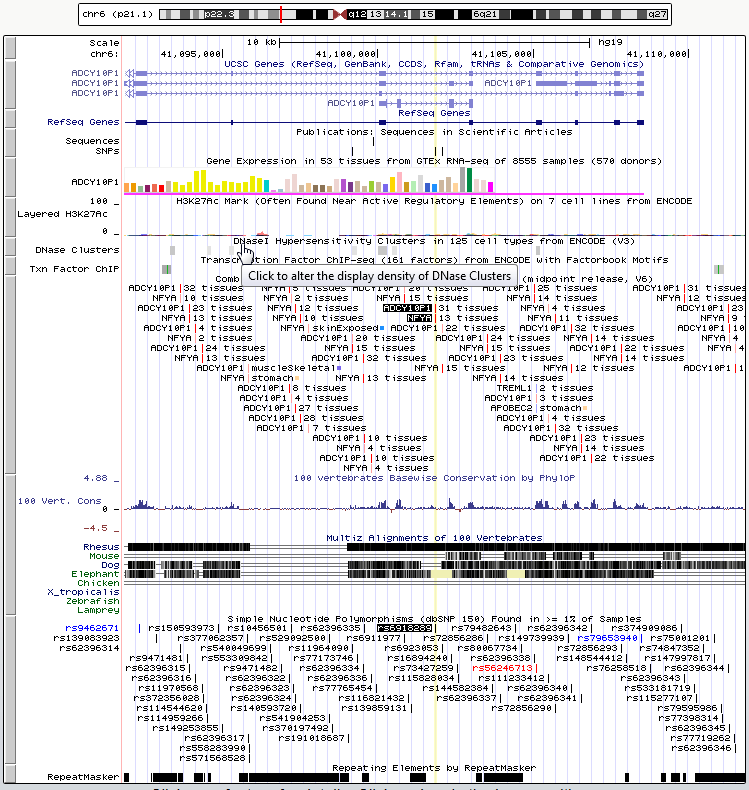


Additional analysis of functional inference was performed with RegulomeDB (<http://regulome.stanford.edu/>). Variant of interest is placed in coding region of human genome, therefore no RegulomeDB Score was available. However, analysis of region with UCSC browser (<http://genome.ucsc.edu/index.html>) highlighted **H3K27ac,** modification to DNA packaging protein Histone H3, usually found near active regulatory elements. Moreover, the dense population of variants in this regions also indicated that rs6918289 is located in an important regulatory region (Sup. Figure 4).
